# Green Nanotechnology: Naturally Sourced Nanoparticles as Antibiofilm and Antivirulence Agents Against Infectious Diseases

**DOI:** 10.1155/ijm/8746754

**Published:** 2025-02-24

**Authors:** Habiba lawal, Shamsaldeen Ibrahim Saeed, Mohammed Sani Gaddafi, Nor Fadhilah Kamaruzzaman

**Affiliations:** ^1^Nanotechnology in Veterinary Medicine (NanoVet) Research Group, Faculty of Veterinary Medicine, University Malaysia Kelantan, Pengkalan Chepa, Kelantan, Malaysia; ^2^Department of Public Health, Ministry of Animal Health, Husbandry and Fisheries, Birnin Kebbi, Kebbi State, Nigeria; ^3^School of Food and Biological Engineering, Jiangsu University, Zhenjiang, Jiangsu, China; ^4^Department of Microbiology, Faculty of Veterinary Science, University of Nyala, Nyala, Sudan

**Keywords:** Ag, infectious disease, nanoparticle, natural sources, ZnO

## Abstract

The escalating threat of infectious diseases, exacerbated by antimicrobial resistance (AMR) and biofilm formation, necessitates innovative therapeutic strategies. This review presents a comprehensive exploration of the potential of nanoparticles synthesized from natural sources, including plant extracts, microbial products, and marine compounds, as antimicrobial agents. These naturally derived nanoparticles demonstrated significant antibiofilm and antivirulence effects, with specific examples revealing their capacity to reduce biofilm mass by up to 78% and inhibit bacterial quorum sensing by 65%. The integration of bioactive compounds, such as polyphenols and chitosan, facilitates nanoparticle stability and enhances antimicrobial efficacy, while green synthesis protocols reduce environmental risks. Notably, the review identifies the potential of silver nanoparticles synthesized using green tea extracts, achieving 85% inhibition of polymicrobial growth in vitro. Despite these promising results, challenges such as standardization of synthesis protocols and scalability persist. This study underscores the transformative potential of leveraging naturally sourced nanoparticles as sustainable alternatives to conventional antimicrobials, offering quantitative insights for their future application in combating mono- and polymicrobial infections.

## 1. Introduction

In the face of escalating mono- and polymicrobial diseases, conventional antibiotic therapies are increasingly proving inadequate, necessitating a paradigm shift toward innovative and sustainable solutions [1]. The emergence of biofilm-associated infections and the growing prevalence of antibiotic-resistant strains underscore the urgency of identifying novel therapeutic strategies [2]. The rise of antibiotic resistance has become a formidable challenge, with estimates suggesting that by 2050, antibiotic-resistant infections could cause 10 million deaths annually [3]. Concurrently, biofilm formation by pathogens also has been identified as one of the major contributors to treatment failure, as these biological structures confer enhanced resistance to antibiotics [4]. The lack of antibiotic development by the pharmaceutical companies has worsened the situation, as there are fewer available options for drugs in the pipeline. Therefore, it is important to find an alternative to the existing antibiotic. The progress of material sciences has opened the possibility of utilizing nanosize material in biological applications, including as one of the potential treatments for infection. Nanomaterials, commonly referred to as nanoparticles, are materials with a size range between 1 and 100 nm, according to the International Organization for Standardization (ISO) (ISO 2008). Nanoparticles are larger than a single atom or even small groups of atoms [5]. Nanoparticles have emerged as versatile candidates for combating infections due to their unique physicochemical properties and the ability to interact with microbial cells at the nanoscale [6]. Nanoparticles exhibit antimicrobial activities via several mechanisms, which include sharp incisions to the bacteria, wrapping on the bacterial surface, and causing oxidative stress to the bacteria, thus promoting cell death [7, 8]. The nanoparticles are commonly chemically synthesized, and thus, the process requires exposure to concentrated chemicals; for example, graphene oxide synthesis using the Hummers method requires the use of concentrated hydrochloric acid, leaving a potential residue on the synthesized material. Also, the synthesis of the synthetic nanoparticle may involve the following procedure, for example, vapor deposition gas phase, which can potentially result in human exposure through various routes, namely, inhalation, dermal absorption, or ingestion [9].

Thus, exploring a natural method to synthesize a nanoparticle would be important to reduce the possibility of chemical residuals in the material. Numerous studies have highlighted the potential of plant extracts, microbial products, and marine compounds in the green synthesis of nanoparticles [6]. Such an approach not only aligns with the growing emphasis on sustainable practices but also harnesses the inherent antimicrobial properties of these natural materials. This review manuscript is a modified version of our previous article [10], which has been published as a preprint at 10.22541/au.172192045.50188569/v1, and aims to provide a comprehensive analysis of the antibiofilm and antivirulence properties of nanoparticles synthesized from naturally derived materials. By exploring the intricate mechanisms through which these nanoparticles combat microbial pathogenicity, we endeavor to shed light on their potential as effective agents against mono- and polymicrobial diseases.

## 2. Monomicrobial Diseases

Monomicrobial diseases are infections caused by a single species of microorganism. In these cases, a specific pathogen is responsible for the infection, and the disease manifestations are primarily associated with the characteristics of that particular microbe [11]. Examples of monomicrobial diseases include streptococcal pharyngitis (caused by *Streptococcus pyogenes*), tuberculosis (caused by *Mycobacterium tuberculosis*), and urinary tract infections (often caused by *Escherichia coli*). In these infections, a single pathogen is predominantly responsible for the clinical symptoms [11]. Diagnosing monomicrobial diseases typically involves identifying the specific pathogen through laboratory tests, such as cultures, molecular assays, or serological tests. Treatment strategies are often tailored to target the identified microorganism, and antimicrobial therapy is directed against that specific pathogen [11]. However, factors such as the development of microbial resistance, evasion of the host immune response, and the formation of microbial reservoirs could contribute to the failure of the treatment (Satish [12]).

## 3. Polymicrobial Diseases

Polymicrobial diseases involve infections where multiple microbial species coexist and contribute to the disease process. The interaction between different microorganisms can lead to complex clinical presentations, with each microbe potentially influencing the behavior and virulence of others [13]. Polymicrobial diseases are diverse and can affect various body sites. Chronic wounds, diabetic foot ulcers, and periodontal infections are examples of polymicrobial diseases, where a combination of bacteria, fungi, and sometimes viruses may be involved [14]. Polymicrobial nature is also observed in certain respiratory tract infections and intra-abdominal infections [13]. Diagnosing polymicrobial diseases can be challenging due to the presence of multiple microorganisms. Advanced diagnostic techniques, including molecular assays and metagenomic sequencing, are increasingly used to identify diverse microbial populations [15]. Treatment strategies often involve a broader spectrum of antimicrobials to target the different pathogens involved. Additionally, addressing the polymicrobial nature may require a multidisciplinary approach, including surgical interventions and wound care in the case of chronic wounds [16]. Interactions between different microbial species in polymicrobial infections can be complex [17]. The presence of multiple microorganisms with different susceptibilities to antimicrobials increases the risk of treatment resistance [18].

## 4. Biofilm Associated Infection

**Figure 1:**
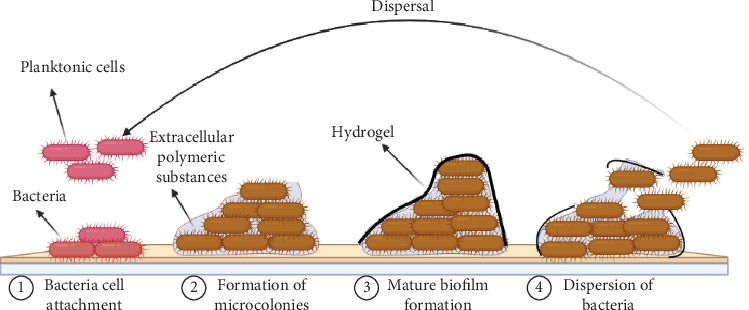
Steps in biofilm formation. The image was created using BioRender.com

Bacterial infections are frequently associated with biofilm formation, which involves a structured community of microorganisms embedded within a self-produced protective matrix [13]. Biofilms can enhance resistance to antimicrobial agents and the host immune response, posing challenges for treatment [19]. Biofilms are formed in a multistep process that involves cell attachment to the surface, adhesion between cells and surface, and formation of extracellular matrix, which are the key factors that protect the bacteria from targeting by antimicrobial therapy, host defense systems, and environmental stress. Biofilms may be developed in any part of the body, such as the respiratory tract, urogenital tract, oral cavity, and udder, as well as in abiotic surfaces such as medical devices. Biofilm-related infections are particularly chronic and characterized by the persistence of the microorganisms. The chronic character of biofilm infections could be associated with a subpopulation of cells located inside them, known as “persister cells” [20].  Figure 1 illustrates the steps of biofilm formation.

## 5. Antimicrobial Effect of Nanoparticle

**Figure 2:**
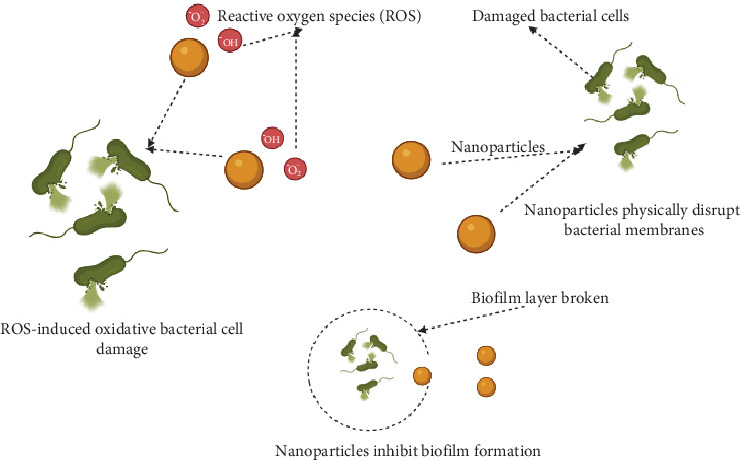
Antimicrobial mechanisms of nanoparticles. The image was created using BioRender.com

Nanoparticles, with their unique physicochemical properties, hold promise in addressing challenges associated with polymicrobial biofilms [21]. Engineered nanoparticles can disrupt biofilm structures, enhance drug delivery, and exhibit antimicrobial effects against multiple species within the biofilm [21]. Nanoparticles with broad-spectrum antimicrobial properties, such as silver nanoparticles, may be particularly useful in polymicrobial infections [22]. These nanoparticles can simultaneously target multiple microbial species, potentially overcoming the complexity of mixed infections [23, 24]. Additionally, combining nanoparticle-based therapies with traditional antimicrobials may offer synergistic effects against polymicrobial infections [25]. Nanoparticles can enhance the efficacy of existing drugs and help overcome challenges associated with diverse microbial populations [26]. Nanoparticles, such as metallic nanoparticles like silver and copper, possess intrinsic properties that enable them to disrupt the biofilm matrix [27]. They can penetrate the extracellular polymeric substances (EPSs) that constitute the matrix, leading to the destabilization and breakdown of the biofilm structure [28]. Certain nanoparticles, including zinc oxide and titanium dioxide, can generate reactive oxygen species (ROS) when exposed to light. ROS exhibit potent antimicrobial effects, inducing oxidative stress within biofilm cells and damaging their cellular components [29]. Additionally, metallic nanoparticles have inherent antibacterial properties, and when incorporated into materials or applied as coatings, they can prevent biofilm formation [30]. These nanoparticles disrupt bacterial cell membranes, interfere with cellular processes, and inhibit the adhesion of microorganisms to surfaces [31]. Silver nanoparticles are among the most studied for their potent antimicrobial and antibiofilm properties [32]. They can disrupt biofilm matrices, inhibit bacterial adhesion, and induce microbial cell death. The versatility of silver nanoparticles allows for incorporation into various materials, making them effective coatings for medical devices [33]. Copper nanoparticles exhibit antimicrobial effects by releasing copper ions, disrupting bacterial cell membranes, and interfering with cellular processes [34]. These properties make copper nanoparticles effective in preventing biofilm formation on surfaces, including those of medical implants [35]. Zinc oxide nanoparticles possess photoactivated antimicrobial properties, generating ROS when exposed to light [36]. This capability makes them effective in disrupting biofilms on surfaces exposed to light, such as wound dressings or catheters [37]. Zinc oxide nanoparticles also exhibit antiadhesive properties, preventing bacterial attachment [37]. Chitosan, derived from marine sources, has shown promise in preventing biofilm formation. Chitosan nanoparticles disrupt the EPS matrix, inhibit quorum sensing (QS), and exhibit antibacterial effects. Their biocompatibility and biodegradability contribute to their potential in biomedical applications [38]. Nanoparticles can also interfere with bacterial communication systems, such as QS, which regulates biofilm formation [39]. QS is a cell-to-cell communication mechanism utilized by bacteria to coordinate collective behaviors, such as biofilm formation, virulence factor production, and antibiotic resistance [39]. This process involves the production, release, and detection of signaling molecules called autoinducers, which enable bacteria to sense their population density and regulate gene expression accordingly [39]. Nanoparticles have emerged as promising anti-QS agents due to their ability to interfere with these signaling pathways [40]. For instance, silver nanoparticles have been shown to inhibit the production of autoinducers, thereby reducing virulence factor expression in *Pseudomonas aeruginosa* [41]. Similarly, chitosan nanoparticles disrupt QS biofilm formation by inhibiting the synthesis of signaling molecules [42]. These mechanisms highlight the potential of nanoparticles to combat bacterial infections by targeting QS systems without imposing selective pressure for resistance development. By disrupting QS, nanoparticles can inhibit the coordinated behavior of bacteria within biofilms, reducing their virulence and persistence [40].  Figure 2 illustrates the antimicrobial activity of nanoparticles.

## 6. Antivirulence Effects of Nanoparticles

Bacterial virulence refers to the ability of bacteria to cause disease in a host organism. Virulence factors are molecular components or strategies that contribute to the pathogenicity of bacteria, allowing them to colonize, invade, and evade host defenses. Antivirulence approaches aim to disarm bacteria by targeting these virulence factors [43]. Certain nanoparticles can neutralize bacterial toxins, which are key virulence factors. For example, macrophage-like nanoparticles can bind to and sequester toxins, preventing them from exerting their damaging effects on host cells [44]. Silver and iron nanoparticles, known for their antimicrobial properties, also exhibit antivirulence effects. They can interfere with QS, a mode of bacterial communication, reduce biofilm formation, and inhibit the production of virulence factors [41]. Additionally, they have been explored for their ability to inhibit bacterial motility, reducing the ability of bacteria to colonize and invade host tissues [45]. Gold nanoparticles have shown the potential to inhibit bacterial adhesion and biofilm formation by interfering with the binding of bacterial adhesins to host cells, disrupting the initial stages of infection [46, 47]. Polymeric nanoparticles, including those made from biocompatible materials like chitosan or poly(lactic-co-glycolic acid) (PLGA), can be designed to deliver antivirulence agents. These nanoparticles offer controlled release and targeted delivery to disrupt virulence mechanisms [48].

## 7. Naturally Derived Materials in Nanoparticle Synthesis

### 7.1. Plant Extracts

One of the most explored avenues in the green synthesis of nanoparticles involves the use of plant extracts rich in bioactive compounds [49]. Various plant species have been investigated for their potential to reduce metal ions and facilitate the synthesis of nanoparticles. For instance, the polyphenols present in green tea extracts have demonstrated efficacy in the formation of silver nanoparticles, imparting them with antimicrobial properties [50]. Similarly, extracts from medicinal plants such as neem (*Azadirachta indica*) and turmeric (*Curcuma longa*) have been harnessed for the synthesis of nanoparticles with significant antibacterial and antifungal activities [51].

### 7.2. Polyphenol–Plant Extract

**Figure 3:**
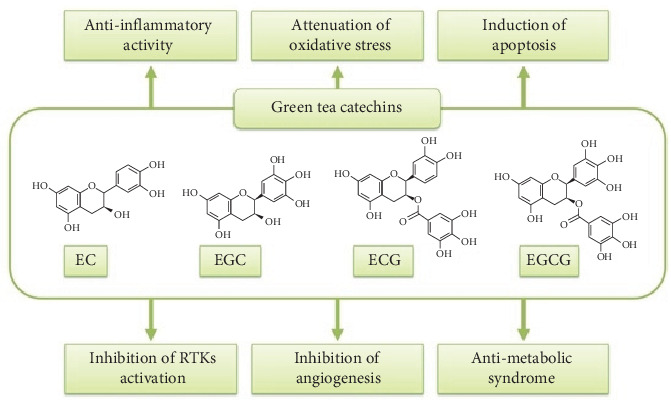
The biological activities of catechins extracted from green tea, adapted from Shirakami and Shimizu [56].

Polyphenols, a diverse group of naturally occurring compounds found in plants, have garnered considerable attention for their role in the green synthesis of nanoparticles [52]. Polyphenols play a pivotal role in the reduction and stabilization of metal ions during nanoparticle synthesis [53]. One good example of a plant with a high concentration of polyphenols is green tea. Green tea extracts, derived from the leaves of *Camellia sinensis*, have gained significant attention as potent sources of polyphenols, particularly catechins, for the green synthesis of silver nanoparticles [50]. Catechins, a subclass of flavonoids found abundantly in green tea, are key contributors to the bioactivity of green tea extracts in nanoparticle synthesis [54]. Epigallocatechin gallate (EGCG), epicatechin gallate (ECG), epigallocatechin (EGC), and epicatechin (EC) are among the major catechins present in green tea, each exhibiting distinct antioxidant and reducing properties [55] (Figure 3). These catechins play a pivotal role in the reduction of metal ions and subsequent nanoparticle formation [57]. The unique chemical composition of green tea imparts remarkable reducing and stabilizing capabilities, making it a valuable resource in nanomaterial synthesis [50]. Silver nanoparticles synthesized using green tea extracts exhibit pronounced antimicrobial effects against the following pathogens: *Enterococcus faecium*, *Staphylococcus aureus*, *Klebsiella pneumoniae*, *Acinetobacter baumannii*, *Pseudomonas aeruginosa*, and *Enterobacter* spp. [58]. The synergistic action of catechins and the inherent properties of the metal nanoparticles contribute to disrupting the microbial cell membranes and inhibiting growth [59]. The multifaceted mechanisms of action include disruption of cell membranes, interference with microbial enzymes, and induction of oxidative stress, collectively contributing to the antimicrobial effects of these nanoparticles [60].

Nanoparticle synthesis mediated by polyphenols involves the reduction of metal ions by complex redox reactions. Polyphenols donate electrons to the metal ions, leading to their reduction and subsequent nucleation and growth of nanoparticles [61]. The functional groups in polyphenols, such as hydroxyl and carbonyl groups, actively participate in the reduction process [62]. Additionally, the ability of polyphenols to chelate metal ions aids in controlling the size and morphology of the resulting nanoparticles [63]. The redox-active catechins in green tea extracts facilitate the reduction of metal ions through electron donation, leading to the nucleation and growth of nanoparticles [64]. The hydroxyl groups in catechins actively participate in the reduction process, resulting in the formation of stable nanoparticles with controlled sizes and shapes [65]. The utilization of polyphenol-rich extracts aligns with the principles of green and sustainable synthesis [66]. Unlike conventional chemical methods that may involve toxic reagents, the use of plant extracts reduces the environmental impact of nanoparticle synthesis, offering a more ecofriendly alternative [67]. Also, the nature of green tea extracts offers a degree of control over the size and morphology of the synthesized nanoparticles [65]. The concentration of catechins, reaction temperature, and pH play crucial roles in determining these characteristics [57]. The inherent antioxidant properties of catechins also contribute to the prevention of nanoparticle agglomeration, ensuring a well-defined and stable nanomaterial product [68].

### 7.3. Medicinal Plant Extracts

Medicinal plants, with their diverse array of bioactive compounds, offer a treasure trove for nanoparticle synthesis [69]. Extracts from plants such as neem (*Azadirachta indica*) and turmeric (*Curcuma longa*) have demonstrated remarkable potential in the green synthesis of nanoparticles with strong antimicrobial activities [70]. Neem, a medicinal plant native to South Asia, is widely recognized for its diverse therapeutic properties and potential applications [71]. Neem, rich in a variety of bioactive compounds, such as terpenoids, flavonoids, limonoids (azadirachtin), nimbin, nimbidin, quercetin, and alkaloids, has gained prominence as a potent resource for the green synthesis of nanoparticles [72, 73]. These unique pharmacological properties make them suitable for a wide range of applications [74]. Neem extracts have been successfully employed in the green synthesis of silver and gold nanoparticles [75]. The reducing and stabilizing capabilities of neem compounds play a crucial role in the conversion of metal ions into stable nanoparticles [76]. The green synthesis approach using neem extracts is considered environmentally friendly and sustainable [77].

Turmeric (*Curcuma longa*), a perennial herb native to South Asia, has been widely recognized for its culinary and medicinal uses [78]. The primary bioactive compound in turmeric is curcumin, a polyphenol known for its anti-inflammatory, antioxidant, and antimicrobial properties [79]. Turmeric extracts, particularly those enriched with curcumin, have gained significance in the green synthesis of nanoparticles [80]. The key bioactive compound in turmeric extracts is curcumin, a polyphenol with pronounced pharmacological effects [81]. Curcumin's unique structure and chemical properties make it an ideal candidate for nanoparticle synthesis, particularly in the green synthesis approach [82]. Nanoparticles synthesized using medicinal plant extracts often exhibit potent antimicrobial properties [83]. The interactions between the bioactive compounds from medicinal plants and the synthesized metal nanoparticles contribute to disrupting microbial membranes, inhibiting microbial growth, and demonstrating antimicrobial activities against a broad spectrum of microorganisms, including bacteria and fungi [70, 84]. Beyond their antimicrobial effects, nanoparticles derived from medicinal plant extracts, especially those containing anti-inflammatory compounds, show promise for therapeutic applications [83]. The anti-inflammatory properties of these nanoparticles position them for potential use in conditions characterized by inflammation and oxidative stress [85]. The inherent antioxidant properties of curcumin contribute to the antioxidant effects of nanoparticles synthesized with turmeric extracts [86]. These nanoparticles can scavenge free radicals and mitigate oxidative stress, making them valuable in conditions associated with increased oxidative damage [87]. The antimicrobial and anti-inflammatory properties of turmeric extract–mediated nanoparticles contribute to their potential in wound healing and tissue regeneration. These nanoparticles can aid in preventing infections, reducing inflammation, and promoting the regeneration of damaged tissues, making them valuable in the field of regenerative medicine [88]. The biocompatibility of nanoparticles synthesized using turmeric extracts, combined with the controlled release properties of curcumin, positions them as promising carriers for drug delivery [89]. The controlled and sustained release of therapeutic agents from these nanoparticles enhances their efficacy in targeted drug delivery applications [90]. The active components in medicinal plant extracts, such as polyphenols, flavonoids, and alkaloids, participate in redox reactions, facilitating the reduction of metal ions and subsequent nanoparticle formation [65]. The rich chemical diversity of these plant extracts contributes to the controlled growth and stabilization of nanoparticles, influencing their size, shape, and surface characteristics [91]. The bioactive compounds in neem extracts, particularly terpenoids and flavonoids, serve as potent reducing agents during nanoparticle synthesis [92]. These compounds donate electrons, leading to the reduction of metal ions and subsequent formation of nanoparticles [91]. The presence of secondary metabolites in neem extracts also contributes to the stabilization of nanoparticles [93]. The synthesis of nanoparticles using turmeric extracts, primarily enriched with curcumin, involves intricate redox reactions [82]. Curcumin, with its polyphenolic structure, serves as a potent reducing agent, facilitating the reduction of metal ions [94]. The phenolic hydroxyl groups actively participate in electron donation, leading to the nucleation and growth of nanoparticles [95]. Additionally, curcumin acts as a capping agent, contributing to the stability and controlled size distribution of the synthesized nanoparticles [82].

The use of neem extracts aligns with the principles of green and sustainable synthesis [96]. The mild conditions employed in the green synthesis process reduce the environmental impact compared to traditional chemical methods [67]. The ecofriendly nature of neem-derived nanoparticles positions them favorably for various biomedical applications [97]. The biocompatibility of turmeric-derived nanoparticles, attributed to the natural origin of curcumin, makes them suitable for drug delivery applications [79]. The controlled release of therapeutic agents from these nanoparticles, combined with the inherent pharmacological effects of curcumin, enhances their efficacy in targeted drug delivery [98].

Turmeric extracts, enriched with curcumin, have been effectively utilized in the green synthesis of nanoparticles, including metallic nanoparticles like gold and silver [80]. The reducing and capping properties of curcumin play a crucial role in the conversion of metal ions into stable nanoparticles, offering a sustainable and ecofriendly alternative to traditional synthesis methods [82].

### 7.4. Microbial Products

Microorganisms, including bacteria and fungi, produce a diverse array of metabolites that can serve as reducing and stabilizing agents in nanoparticle synthesis [72]. Microbial synthesis offers advantages such as scalability, cost-effectiveness, and the ability to produce nanoparticles under mild conditions [64]. Bacterial EPSs and fungal exopolysaccharides, for instance, have been employed in the synthesis of metal and metal oxide nanoparticles with antimicrobial properties [99]. The use of microbial products not only provides a sustainable alternative to chemical synthesis methods but also opens avenues for tailoring nanoparticle properties through genetic manipulation of the microbial hosts [100]. Microbial products, such as bacterial EPSs, have been studied for nanoparticle synthesis. EPS, also often referred to as the extracellular matrix, is a complex mixture of polysaccharides, proteins, nucleic acids, and lipids produced by bacteria [12]. Bacterial EPS is highly diverse in composition, varying between bacterial species and environmental conditions [101]. Fungi, too, contribute to nanoparticle synthesis through the production of exopolysaccharides. Polymers secreted by fungi have been utilized in the green synthesis of metal and metal oxide nanoparticles [102]. Fungal exopolysaccharides act as effective reducing agents, and their chemical composition influences the size and shape of the resulting nanoparticles [103]. Fungal EPS is a diverse mixture of polysaccharides, proteins, lipids, and other biopolymers, with the specific composition varying depending on the fungal species and growth conditions. Common fungal EPS include glucans, mannans, and other complex sugar-based polymers [104].

EPS has been explored for its role in the green synthesis of nanoparticles, particularly metallic nanoparticles such as silver and gold [105]. The inherent reducing and capping properties of EPS contribute to the formation of stable nanoparticles [69]. Nanoparticles synthesized using bacterial and fungal EPS have exhibited pronounced antimicrobial properties [106]. The interaction of these nanoparticles with microbial cell membranes disrupts cellular structures, leading to the inhibition of microbial growth [106]. This antimicrobial efficacy positions them as potential candidates for various biomedical applications, including coatings for medical devices and wound dressings [16]. The functional groups on polysaccharides and proteins of bacterial and fungal EPS can act as potent reducing and stabilizing agents in the green synthesis of nanoparticles [12]. The mechanisms underlying the synthesis involve the interaction of metal ions with the functional groups (hydroxyl and amino groups) present in bacterial EPS (Figure 3) [107, 108]. The electron-donating properties of these functional groups facilitate the reduction of metal ions to nanoparticles [109]. The complex matrix of bacterial EPS also acts as a stabilizing agent, preventing the agglomeration of nanoparticles and ensuring their stability [33]. Bacterial EPS also influences the size and shape of the resulting nanoparticles [99]. The controlled growth of nanoparticles within the EPS matrix contributes to the uniformity of size, a critical factor in determining the properties and applications of the synthesized nanoparticles [110].

**Figure 4:**
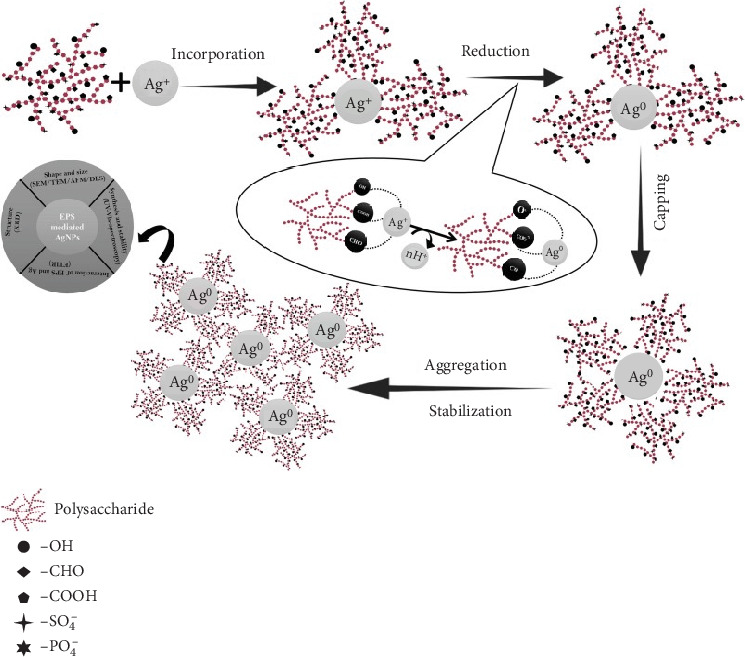
Mechanisms of bacterial EPS-mediated nanoparticle synthesis, adapted from Dey et al. [113].

The use of bacterial and fungal EPS in nanoparticle synthesis aligns with the principles of environmental sustainability [111]. The EPS-mediated nanoparticle synthesis can be conducted under mild and environmentally friendly conditions, reducing the ecological footprint associated with traditional chemical synthesis methods [112] (Figure 4).

### 7.5. Marine Compounds

The rich biodiversity of marine environments has spurred interest in utilizing marine-derived compounds for nanoparticle synthesis [114]. Bioprospecting involves the exploration of marine organisms, such as algae, sponges, and microorganisms, for bioactive compounds with potential applications in various fields, including nanotechnology [115]. The rich biodiversity of marine ecosystems provides a source of unique molecules that can serve as reducing and stabilizing agents in nanoparticle synthesis [114]. This diversity extends from microscopic plankton to large marine mammals, providing a rich source of bioactive compounds with novel chemical structures and functionalities [116].

Algae, including microalgae and macroalgae, produce a wide range of bioactive compounds such as polysaccharides, polyphenols, and pigments [117]. These compounds have been utilized in the green synthesis of nanoparticles, particularly metallic nanoparticles. Chitosan, a polysaccharide derived from chitin, is abundantly found in the exoskeletons of crustaceans and insects [118]. Chitosan has been widely used in nanoparticle synthesis due to its biocompatibility, biodegradability, and unique chemical properties. Marine-derived chitosan offers a sustainable alternative for the synthesis of chitosan-coated nanoparticles [119].

Marine bacteria and fungi are essential components of marine ecosystems and contribute significantly to the production of bioactive compounds [120]. These microorganisms have adapted to extreme conditions in the marine environment, and their metabolites often exhibit unique properties [121]. Sponges and other marine invertebrates are prolific sources of secondary metabolites with diverse chemical structures [122]. These compounds, including alkaloids, terpenoids, and peptides, have demonstrated various bioactivities. Polysaccharides derived from marine sources, such as fucoidan and carrageenan, have been explored for their role in nanoparticle synthesis and drug delivery [123]. These marine polysaccharides can serve as both reducing agents and stabilizing agents, contributing to the development of nanocarriers for drug delivery applications [124].

Utilizing marine compounds in nanoparticle synthesis aligns with principles of environmental sustainability [64]. Marine resources offer a renewable and often underexplored pool of bioactive compounds that can be harnessed without significant ecological impact [125]. The sustainable use of marine-derived compounds contributes to the development of ecofriendly nanotechnologies [114].

## 8. Challenges in Utilizing Natural Products for Nanoparticle Synthesis

The utilization of natural products for nanoparticle synthesis has been a promising strategy considering the ecofriendly approach required for the process. However, there are several important challenges that require further investigations to ensure the nanoparticles can be synthesized at a larger scale. Plant extracts offer a wealth of advantages, but challenges include standardization of extraction methods and variability in the active compound content, posing considerations for optimization [50]. For example, while polyphenol-rich extracts offer numerous advantages, challenges such as batch-to-batch variability and the need for standardized extraction protocols remain to produce a high concentration of product [126]. Optimization of synthesis conditions, including the concentration of polyphenols and reaction parameters, is essential to ensure reproducibility and scalability for practical applications [127].

Additionally, challenges in utilizing bacterial and fungal EPS for nanoparticle synthesis include optimizing culture conditions to enhance the yield of EPS with desired properties, addressing potential variability in EPS composition, and ensuring reproducibility [99, 128]. Finally, challenges in bioprospecting include the need for sustainable harvesting practices, ethical considerations related to biodiversity conservation, and the development of fair and equitable benefit-sharing mechanisms [129]. Addressing these challenges is crucial to ensure the responsible and sustainable exploration of marine resources [130]. Also, challenges in harnessing marine compounds for nanoparticle synthesis include the optimization of extraction methods and the identification of specific compounds with optimal reducing and stabilizing capabilities [64]. While nanoparticles offer promising antibiofilm properties, assessing their biocompatibility and potential cytotoxic effects is crucial [131]. Understanding the balance between antimicrobial efficacy and safety is essential for the development of clinically relevant antibiofilm strategies [132]. As with any antimicrobial agent, the potential for microbial adaptation and the development of resistance to nanoparticles exist [15]. Research efforts must continue to explore strategies that minimize the likelihood of resistance and enhance the long-term efficacy of antibiofilm nanoparticles [133]. The synergistic approach of combining nanoparticles with other antimicrobial agents or therapies may enhance their antibiofilm effects [21]. This approach can target different aspects of biofilm formation and reduce the risk of resistance development [134]. Ensuring the specificity and selectivity of nanoparticles for bacterial virulence factors is crucial [135]. As such, designing nanoparticles that specifically target pathogenic bacteria without affecting beneficial microbes in the host is a challenge that researchers are actively addressing [136]. As with any therapeutic approach, there is a potential for bacteria to adapt and develop resistance [18]. Research efforts should focus on understanding the mechanisms underlying resistance and developing strategies to minimize its occurrence [137]. Translating antivirulence strategies from in vitro studies to in vivo efficacy poses challenges [138]. Factors such as nanoparticle stability, biodistribution, and the host immune response need to be considered for successful application in living organisms.

## 9. Conclusion and Future Direction

This review underscores the immense potential of nanoparticles synthesized from naturally derived materials, including plant extracts, microbial products, and marine compounds, as transformative agents in combating infectious diseases. Key findings reveal that these nanoparticles not only exhibit significant antimicrobial and antibiofilm activities but also address critical challenges associated with antimicrobial resistance (AMR) and polymicrobial infections. Innovative approaches such as green synthesis protocols using bioactive compounds from natural sources align with sustainability principles while enhancing nanoparticle stability and functional efficacy.

We hypothesize that the multifunctional properties of these nanoparticles, such as targeted biofilm penetration, ROS generation, and QS disruption, represent a paradigm shift in antimicrobial therapy. By leveraging these mechanisms, future applications could extend beyond infection control to targeted drug delivery, wound healing, and regenerative medicine. Additionally, the integration of green synthesis methods presents an innovative avenue to mitigate environmental and biocompatibility concerns associated with conventional nanoparticle synthesis.

Key improvements highlighted in this review include advancements in nanoparticle stability, scalability of green synthesis processes, and enhanced antimicrobial efficacy through the synergistic use of natural bioactive compounds. Notably, plant-derived nanoparticles demonstrated the potential to reduce biofilm mass by up to 78%, offering tangible benefits for addressing chronic infections.

In the future direction, we envision a multidisciplinary effort to standardize synthesis protocols, optimize nanoparticle design for clinical applications, and expand the exploration of underutilized natural sources such as marine ecosystems. Future work should also focus on elucidating the molecular interactions of nanoparticles with microbial and host systems to refine their specificity and minimize unintended ecological impacts. By addressing these priorities, naturally synthesized nanoparticles could revolutionize antimicrobial therapies, marking a critical step toward overcoming the global AMR crisis.

## Data Availability

The corresponding authors have made all of the study's data available upon request. They are all included in this manuscript.
